# An Impairment of Prospective Memory in Mild Alzheimer’s Disease: A Ride in a Virtual Town

**DOI:** 10.3389/fpsyg.2019.00241

**Published:** 2019-02-12

**Authors:** Grégory Lecouvey, Alexandrine Morand, Julie Gonneaud, Pascale Piolino, Eric Orriols, Alice Pélerin, Laurence Ferreira Da Silva, Vincent de La Sayette, Francis Eustache, Béatrice Desgranges

**Affiliations:** ^1^Normandie Université, UNICAEN, PSL Universités Paris, EPHE, INSERM, U1077, CHU de Caen, Neuropsychologie et Imagerie de la Mémoire Humaine, Caen, France; ^2^Laboratoire Mémoire et Cognition, Institut de Psychologie and Centre de Psychiatrie et Neurosciences, INSERM S894, Université Paris Descartes, Paris, France

**Keywords:** intentions, virtual reality, Alzheimer’s disease, prospective component, retrospective component, event-based, time-based

## Abstract

**Objective:** Research suggests that prospective memory (PM) is impaired from the very early stages of Alzheimer’s disease (AD). We sought to further characterize this impairment in patients with mild AD, using a virtual reality (VR) task to provide ecological assessment of PM.

**Methods:** Fifteen cognitively normal older individuals (76.47 years old ± 4.14, MMSE: 28.80 ± 1.21), and 17 patients with mild AD (79.29 years old ± 4.45, MMSE: 22.82 ± 2.83) were asked to recall the prospective and retrospective components of seven intentions in a virtual town task. Six intentions were event-based, where the prospective cue was either highly (three intentions) or weakly (three intentions) associated with the retrospective component. The remaining intention was time-based. All participants completed a neuropsychological assessment of episodic memory, semantic memory and executive functioning. Non-parametric tests were used to compare the two groups on the different intentions types and components. Correlations between cognition and PM scores were then realized to further understand the cognitive correlates of the PM impairment in patients with AD.

**Results:** Overall, patients with Alzheimer disease recalled fewer intentions than controls, with the retrospective component and *time-based* intentions being the most challenging for them. The strength of the association between the prospective and retrospective components, however, had no effect on their performance. Event-based PM impairment, as well as deficit in the recall of prospective component correlated with memory and executive functions performance.

**Conclusion:** PM is impaired in AD. Both automatic and controlled processes of PM retrieval are disturbed. This study also confirms the reliability of VR for assessing complex cognitive functions such as PM.

## Introduction

Sporadic Alzheimer’s disease (AD) is a progressive neurodegenerative pathology that mainly affects individuals over 65 years. Despite advances in the analysis of cerebrospinal fluid and neuroimaging biomarkers, neuropsychological assessments remain an essential diagnostic tool in order to detect the earliest cognitive and behavioral critical symptoms of AD. Early diagnosis of AD remains an important issue as it enables caregivers to plan the management of the disease and to provide tailored interventional therapies (Buracchio and Kaye, 2009). Episodic memory impairment is usually described as the key symptom of disease onset and remains a major criterion for the diagnosis of probable AD (McKhann et al., 1984, 2011; Dubois et al., 2014). Forgetfulness of intentions has also been found as an early hallmark of AD (Jones et al., 2006). Nevertheless, prospective memory (PM) assessments are still rare in clinical practice.

Prospective memory is defined as the memory for intentions. It enables us to remember to carry out an action that has been planned for a predefined time in the future, while performing a concurrent activity named *ongoing task* (Einstein and McDaniel, 1990). Retrieving an intention entails (i) remembering at the right moment that something has to be done (prospective component), and (ii) remembering what has to be done (Einstein and McDaniel, 1990). It is usual to distinguish *time-based* (TB) and *event-based* (EB) intentions according to the nature of the stimulus that triggers the retrieval. In this context, a predefined time (e.g., 1:30 pm) or the end of an interval (e.g., in 30 min) triggers TB intention retrieval. Because no clue appears within the environment, remembering TB intentions is suggested to rely on self-initiated processes to monitor time (Craik, 1986). TB intentions are generally assumed to be more challenging to recall than EB ones because their recall relies on the involvement of self-initiated controlled processes to monitor the passage of time (d’Ydewalle et al., 2001). Indeed, monitoring the time while being involved in other (i.e., ongoing) activities requires considerable cognitive resources, including executive functions, attentional resources, and time-estimation abilities. For EB intentions, an external event (prospective cue) that appears within the environment will trigger the intention retrieval (e.g., buy a book of stamps when going past the *post-office*). The retrieval of EB intentions has been suggested to mainly rely on automatic processes. However, according to the *multiprocess theory* of PM (Einstein and McDaniel, 2005), the extent to which cognitive resources are involved in the retrieval of EB intentions depends on the characteristics of the task. Retrieval is thought to be more automatic when the ongoing task requires only limited attentional resources (Einstein et al., 1997), and when the prospective cue is either focal (i.e., when the detection of the cue relies on the same processes as the completion of the ongoing task (Brewer et al., 2010) or distinctive (i.e., easy to distinguish in the environment) (McDaniel and Einstein, 1993). Additional controlled processes will be involved in the opposite conditions. The *reflexive-associative theory* additionally suggests that when the prospective cue and the retrospective component are strongly associated, the mere perception of the prospective cue is sufficient to reflexively trigger the activation of the intention in mind (McDaniel et al., 2004). By contrast, when these elements are weakly associated, retrieval is more challenging because it requires additional controlled processes.

### Prospective Memory Impairment in Alzheimer’s Disease

In studies using subjective questionnaires such as the Prospective and Retrospective Memory Questionnaire (Smith et al., 2000), patients with AD reported at least as many prospective as retrospective failures in everyday life (Ryu et al., 2016). These prospective failures are liable to household accidents, and have been described as more frustrating than retrospective failures by caregivers (Smith et al., 2000).

Huppert and Beardsall (1993) were the first to carry out an experimental study in AD featuring an ecological assessment of PM. Patients with very mild or mild-to-moderate AD were compared to cognitively normal older individuals on the PM subtests of the Rivermead Behavioral Memory Test (Wilson et al., 1985). Surprisingly, while both AD groups performed worse than cognitively normal individuals on the PM subtests, no difference was found between patients with very mild vs. mild-to-moderate AD, suggesting that massive PM impairment occurs in the very early stages of AD, and remains constant thereafter, despite the worsening of the disease.

Huppert et al. (2000) extended these findings in a population-based study in which participants had to remember to put a name and address on an envelope, seal it, and write their initials on the flap when the envelope was given to them. Only 3% of patients with mild dementia successfully completed the task, compared with 54% of cognitively normal older individuals. An increase of 1 point on the retrospective episodic memory test brought 23% additional chances of PM success, suggesting that PM and retrospective episodic memory have several processes in common (but see Jones et al., 2006, for divergent results). Despite showing a major PM impairment, 20% of these participants with AD were living alone.

### Impaired Recall of Event-Based and Time-Based Intentions

Computer-based laboratory experiments have been conducted to investigate the characteristics of the PM impairment in AD. In the first experiment provided by Maylor et al. (2002), participants were asked either to press a key every 3 min (i.e., TB intention) or to verbally report the appearance of an animal (i.e., EB intention) while watching a movie (i.e., ongoing task). Patients with AD performed more poorly than cognitively normal older individuals on the recall of both EB and TB intentions, with a stronger impairment for the recall of TB intentions (Dermody et al., 2016). In their second experiment, Maylor et al. (2002) compared participants’ performance on the recall of EB intentions when the relationship between the prospective cue and retrospective component was either strong (i.e., “when you see a clock, stop the clock”) or weak (i.e., “when you see an animal, stop the clock”). Patients with AD performed equally poorly in both conditions, suggesting that both automatic and controlled processes are impaired in AD. More recently, a study used an ecological approach to assess PM in dementia (Thompson et al., 2010). PM was measured by a computerized board game named Virtual Week where participants had to recall series of time-based and event-based intentions. MCI patients and patients with dementia showed difficulties compared to cognitively normal older individuals, especially for event-based intentions. This study also showed that the utilization of the Virtual Week gives a reliable indicator of PM.

### Impaired Recall of Prospective and Retrospective Components

Studies conducted in the field of normal aging showed that cognitively normal older individuals are less efficient than young individuals on the recall of both prospective and retrospective components of intentions (Zimmermann and Meier, 2006, 2010). In AD, Jones et al. (2006) were the first to investigate separately the recall of these components. In a study conducted over 6 years, data were collected in 650 cognitively normal individuals aged 75 years and over. Neuropsychological assessments were performed at inclusion, and 3 and 6 years later. Forty-six participants who were diagnosed with AD in the course of this follow-up were compared to 188 participants who remained cognitively normal on the recall of one EB intention. They were asked to remind the experimenter to make an important phone call when he announced that all the tests had been completed. If they failed to freely recall the action they had to perform, the authors administered a cued recall test. Free recall was deemed to measure recall of the prospective component of the situation, and cued recall that of the retrospective component. Results showed comparable impairment for the recall of the prospective and retrospective components 3 years before the AD diagnosis, suggesting that episodic memory may be involved not only in the recall of the retrospective component, but also in the recall of the prospective component.

### Virtual Reality: A Step Toward Real-World Evaluation

The above-mentioned studies mostly used classic tools to assess PM. Although these tools have made a major contribution in highlighting and characterizing PM impairment, immersing individuals in complex everyday-life situations may enhance the sensitivity of PM tasks and improve the detection of inconspicuous impairments. Virtual reality (VR), a technology that immerses individuals in non-real environments, creates naturalistic conditions while maintaining a high level of control in experimental tasks (Knight and Titov, 2009). It has notably been used to assess PM in young (Kalpouzos et al., 2010; Trawley et al., 2011), as well as in cognitively normal older individuals (Lecouvey et al., 2017), but never in AD patients. Nevertheless, several studies suggested the relevance and feasibility of such assessment to investigate episodic memory (Plancher et al., 2012b; Widmann et al., 2012; Sauzéon et al., 2014), spatial navigation (Drzezga et al., 2005; Cushman et al., 2008; Zakzanis et al., 2009), and driving abilities in AD patients (see García-Betances et al., 2015, for review).

### Aims of This Study

Prospective memory impairment in AD is widely acknowledged, but some uncertainties remain regarding the characteristics of this impairment. Indeed, few studies have compared the retrieval of EB vs. TB intentions as well as the retrieval of the prospective vs. retrospective components of these intentions and tried to identify the cognitive substrates of such impairment. In order to better characterize AD-related PM impairment, we compared cognitively normal older individuals and patients with mild AD on a VR task in which they were asked to retrieve the prospective and retrospective components of EB and TB intentions. Participants were asked to recall (1) three EB intentions where there was a strong link between the prospective cue and the retrospective component (Link-EB), (2) three EB intentions where there was a weak link between the prospective cue and the retrospective component (NoLink-EB), and (3) one TB intention that had to be repeated over time. We expected cognitively normal older individuals to be more efficient in recalling Link-EB intentions than NoLink-EB and TB intentions, as the former may rely more on automatic processes preserved in normal ageing. By contrast, we expected patients with AD to show similar impairment in Link-EB and NoLink-EB recall because both automatic and controlled processes are reported to be impaired in AD. We expected TB intentions to be even more challenging for them, as these intentions require controlled processes in a larger extent.

In order to better understand the nature of PM deficit in AD, we attempted to differentiate the prospective and the retrospective components for several reasons: first, in daily life, the prospective component could be forgotten even if the retrospective component is still in memory. Reversely, it is still possible to remember that something has to be done but forget what has to be done. Additionally, previous studies (e.g., Simons et al., 2006) highlighted that different cognitive and cerebral substrates are associated with the prospective and retrospective components, suggesting that they are, at least partially, independent and justify a separate assessment of the two components. Our hypothesis was that in AD, the retrieval of the retrospective component is affected before the prospective one because of the early retrospective episodic memory impairment in this disease.

## Materials and Methods

### Participants

We recruited 15 cognitively normal older individuals (71–86 years old) and 17 patients with mild AD (72–86 years old) for this study (Table 1). All participants were native French speakers, had at least 7 years of schooling, and had normal or corrected-to-normal vision. They all held a driver’s license. We ensured that none of the participants had any antecedents of neurological or psychiatric disorders.

**Table 1:** Sample demographics.

	Cognitively normal
	older individuals	AD	*p*
Age (±SD)	76.47 (±4.14)	79.29 (±4.45)	ns
[range]	[71–86]	[72–86]	
Sex ratio (women/men)	10/5	10/7	ns
Years of schooling (±SD)	12.33 (±2.94)	9.67 (±3.39)	^∗^
Beck Depression Inventory (±SD)	3.40 (±3.72)	4.82 (±5.51)	ns
STAI	33.47 (±8.60)	33.35 (±8.33)	ns
MMSE (±SD)	28.80 (±1.21)	22.82 (±2.83)	^∗∗∗^
MDRS (±SD)	140.47 (±3.62)	118.69 (±8.89)	^∗∗∗^


*SD = standard deviation. Mann–Whitney *U* test significance = ^∗^*p* < 0.05; ^∗∗^*p* < 0.01; ^∗∗∗^*p* < 0.001; ns = non-significant.*

Cognitively normal older individuals were screened with the Mattis Dementia Rating Scale (MDRS; Mattis, 1976) and only those who had a normal score were included. Patients with AD were recruited from local memory clinics. They had all received a diagnosis of probable AD based on standard National Institute of Neurological and Communicative Disorders and Stroke, and AD and Related Disorders Association criteria (McKhann et al., 1984). Some of them were under medication for the disease.

Non-parametric tests (Mann–Whitney *U* test and Chi^2^ test) revealed that the two groups were equivalent in terms of age (*z* = –1.65, *p* = 0.10), sex ratio (χ^2^ = 1.192, *p* = 0.55), level of depression, as assessed by the Beck Depression Inventory (*z* = –0.88, *p* = 0.39) and level of anxiety during VR assessed by the State Trait Anxiety Inventory (STAI) (*z* = 0.08, *p* = 0.94). AD patients demonstrated lower levels of education than the cognitively normal older individuals (*z* = 2.25, *p* = 0.02). Analyses were thus run using both the full sample of participants and subsamples matched for level of education. Results were similar using the whole and matched samples. Consequently, results with the whole sample will be presented in the forthcoming analyses. Finally, AD had lower scores on the MMSE (Folstein et al., 1975) (*z* = 4.68, *p* < 0.001) and MDRS (*z* = 4.48, *p* < 0.001). This study was approved by the regional ethics committee (CCP Nord Ouest III) and all participants gave written consent prior participation to the study. All subjects gave written informed consent in accordance with the Declaration of Helsinki.

### Neuropsychological Assessment

In order to gain a clear picture of the patients’ neuropsychological disturbance, and assess how they are related to PM impairment, participants underwent a cognitive assessment of retrospective episodic memory, semantic memory, and executive functions.

Retrospective episodic memory was assessed with the RL-RI16 (Van der Linden et al., 2004; adapted from Grober and Buschke, 1987). This test comprises three free- and cued-recall tests of a series of 16 words, followed by a recognition task of these 16 words from a total of 48 items. After a 20-min interval, participants are asked to perform a delayed free- and cued-recall of the words. Retrospective memory was scored as the sum of correct answers to the three immediate and the delayed free recalls (/64).

*Semantic memory* was assessed using a picture-naming task in which participants had to name 80 pictures (DO80; Deloche and Hannequin, 1997).

*Fluency* was assessed using categorical and lexical fluency tests. In the categorical fluency test, participants had to give as many words corresponding to animal category as possible in 1 min. In lexical fluency test, participants were asked to give as many words as possible starting with a specific letter (e.g., F) in 1 min.

*Executive functions* were assessed using three tests. (1) Inhibition was assessed with the Stroop test (Stroop, 1992). The inhibition score was calculated as the slowdown in response times in the interference condition relative to the naming of colored rectangles condition ([color time – interference time]/interference time) (see Perrotin et al., 2006). (2) Shifting was assessed with the Trail Making Test (TMT; Army Individual Test Battery, 1944). The shifting score corresponds to the time (in seconds) needed to perform Part B of the test minus the time needed to perform Part A, divided by the time needed to complete Part A ([Part B time – Part A time]/Part A time). (3) Planning was assessed using the Zoo Map test (Allain et al., 2005). In this test, participants are given a map of a zoo and instructed to plan the order in which they would visit designated locations. They have to follow a number of rules (e.g., starting at the entrance and finishing at a designated picnic area, using designated paths in the zoo only once). The score (out of 8) reflects planning accuracy, taking into account both the correct order of the visit and any violations of the rules.

Finally, time monitoring was evaluated by a computerized time estimation task adapted from Pastor et al. (1992) and already used (Lecouvey et al., 2017), where participants should estimate the duration of ten periods of time (being either of 15 s, 30 s, or 40 s).

Group comparisons for episodic memory, semantic memory and executive functions were reported in Table 2. They showed that, compared to cognitively normal individuals, AD patients had lower performances on all cognitive tests of the neuropsychological battery, except Stroop interference.

**Table 2:** Comparisons of results for the cognitive assessment of episodic memory, semantic memory, fluency and executive functions between cognitively normal older adults and patients with AD.

	Cognitively normal
	older individuals	AD	*z*	*p*
**Episodic memory**				
RL-RL16 (±SD)	26.30 (±10.04)	10.70 (±7.50)	4.15	^∗∗∗^
**Semantic memory**				
DO80 (±SD)	79.40 (±0.83)	73.57 (±5.14)	4.07	^∗∗∗^
**Fluency**				
Categorical fluency test (±SD)	28.93 (±6.64)	12.79 (±6.15)	4.39	^∗∗∗^
Lexical fluency test (±SD)	26.00 (±7.94)	11.07 (±4.27)	4.53	^∗∗∗^
**Processing speed**				
Stroop reading (±SD)	76.87 (±44.29)	246.90 (±141.90)	–3.51	^∗∗∗^
**Executive functions**				
**Inhibition**				
Stroop interference (±SD)	1.01 (±0.73)	2.00 (±1.83)	–1.51	ns
**Shifting**				
TMT B-(±SD)	102 (±38.65)	117 (±183.72)	–3.20	^∗∗^
**Planning**				
Zoo (±SD)	2.33 (±0.62)	1.44 (±1.09)	2.33	^∗^
**Time estimation**	42.48 (±24.97)	132.50 (±160.40)	–2.35	^∗^


*SD = standard deviation. Mann–Whitney *U* or Chi^*2*^ test significance = ^∗^*p* < 0.05; ^∗∗^*p* < 0.01; ^∗∗∗^*p* < 0.001; ns = non-significant. For information, only eight patients achieved the TMT.*

### Prospective Memory Assessment

The procedure was adapted from Lecouvey et al. (2017).

#### Virtual Environment

**FIGURE 1:**
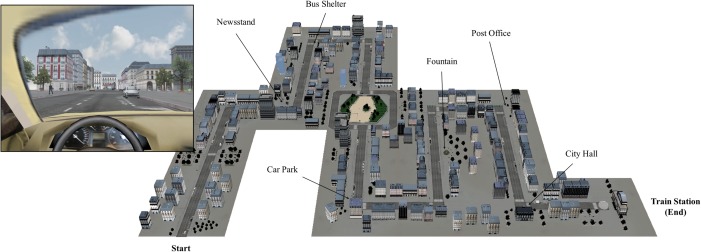
Illustration of the virtual environment including a view from the virtual driving seat during the experiment (left) and a map of the city (right) showing the location of the prospective cues from Lecouvey et al. (2017).

The virtual environment was a virtual town created with an original software (EditoMem and SimulaMem), using Virtools Dev 3.0, and developed by the Memory and Cognition Laboratory of Paris Descartes University (Plancher et al., 2012a,b). Participants navigated in the environment in a virtual car (see Figure 1). This virtual environment was run on a PC laptop computer and projected onto a 180 × 240 cm widescreen. Each participant was tested individually in a quiet room, where he or she was comfortably installed approximately 300 cm from the widescreen. Given the difficulty patients had using equipment during pre-tests (data not shown), the journey through the virtual town was prerecorded. Participants could use two pedals (gas and brake) to drive the car through the city but they could not control the wheel. It was like a car traveling on rails and participants being able to control the speed.

Two cities were proposed. The first environment, used solely in the familiarization phase, was a simple, neutral city with ordinary and similar buildings, some trees and interactive traffic lights. There was only one road (forming a loop), to enable participants to navigate in the environment until they felt fully confident with the device. The second environment was the city of interest. This environment was used twice, once to familiarize the participant with this city of interest (i.e., familiarization with the city of interest) and once for the PM task (see below). This virtual city included various buildings, traffic lights, stores, trees, hoardings, parked cars, pedestrians crossing and corners so that participants had to stop and could not drive too fast. The prospective cues of EB intentions included in the city were located out along the road or in the turns equidistant, so that it would be easy for participants to detect them (i.e., in front of participants at turns or saliently on the side of the road). For the recall of the TB intention, an external clock was positioned so that participants had to deliberately turn the head to look at it to monitor the passage of time. Finally, an urban background noise was added to reinforce participants’ immersion during the experiment.

#### VR Procedure

##### Familiarization with the device

The session began with a familiarization phase, to avoid difficulties arising from the misuse of the device during the PM assessment. Thus, participants were immersed in the first city and informed that they simply had to learn how to use the pedals. They were required to abide usual French road traffic regulations, and traffic lights were placed along their way to prompt them to use the brake. Participants remained immersed in the city until they felt fully confident.

##### Familiarization with the city of interest

This first immersion in the city of interest was intended to ensure that participants would detect and recognize prospective cues in the subsequent PM phase. Participants were told that they would be immersed in a more elaborate city than the previous one, with various buildings, pedestrians and hoardings. This second city had a single road leading to the train station. Participants were asked to move along this road, paying attention to the different features they would encounter. They were again required to abide usual French road traffic regulations. They were informed that they could take as long as they needed to drive through the city.

At the end of their first immersion in the virtual town, participants underwent a recognition test. They had to recognize 14 features of this city out of 22. Eight of these features were distractor items that were not in the present city (i.e., new items: a large road sign, a trash can, a 2D woman, a 2D man, a pedestrian light, a traffic circle, a grid, a supermarket). The remaining 14 items were composed of elements that were present in the city. Six of them were the elements used as prospective cues of the EB intentions in the following parts of the experimentation (i.e., a city hall, a fountain, a post-office, a bus station, a newsstand, a car park; see below) and, eight were neutral items, present in the city but not used in the PM task (i.e., grocery, a roadwork barrier, a tobacco store, a 3D man, a 3D woman, a kebab, a statue, a random building). Feedbacks were given to participants, and the location of any feature they had failed to recognize was shown to them on a map of the city.

##### PM task

After the familiarization phase, participants were informed that they would again be immersed in this virtual city, this time to pick up a friend at the train station (i.e., at the end of this city), and they would have several intentions to fulfill along the way.

##### Encoding

Participants were shown seven intentions on a laptop computer. Each intention was displayed on the screen for 10 s. Six intentions were EB. For half of them, there was a strong link between the prospective cue and the retrospective component (Link-EB; buy a stamp booklet at the post-office, buy a TV program at the newsstand, view bus schedules at the first bus shelter on your way), while for the other half, there was a weak link between them (NoLink-EB; buy a pair of glasses at the fountain, make an appointment at the dentist at the first parking, buy an agenda at the city hall). The remaining intention was a TB one repeated over time (i.e., take medication every 2 min) (see Table 3). Depending on the time it took participants to complete the test, the TB intention could be fulfilled once (i.e., if the participant took less than 4 min to do the task), twice or three times. Then, to ensure that they were correctly encoded, a cued recall test was administered after the intentions had been presented (e.g., “What do you have to do at the post-office?”). Feedbacks were provided for each intention during the cued recall (i.e., correct vs. incorrect). The correct response was directly provided to the participant. For incorrect intentions, unrecalled items were repeated with following cued recall until they were correctly encoded (with a limit of 10 times).

**Table 3:** List of intentions detailing the two components (prospective and retrospective) and the types of intentions (Link-EB, NoLink-EB, TB).

			Order of
	Prospective	Retrospective	appearance
	component	component	in the city
Link-EB	Post-office	Buy a stamp booklet	3
	Newsstand	Buy a TV program	5
	Bus shelter	View bus schedules	4
NoLink-EB	Fountain	Buy a pair of glasses	2
	Parking	Make an appointment at the dentist	6
	City hall	Buy an agenda	1
TB	Every 2 min	Take medication	


##### Storage

There was a 10-min interval between the encoding and retrieval of intentions, filled by the completion of questionnaires.

##### Retrieval

After the 10-min delay, participants were immersed in the virtual city again. They were reminded that they would have to pick up a friend at the train station and fulfill several intentions along the way. To do so, they had to stop the car at the appropriate time or place (i.e., prospective component) and tell the experimenter which action they had to perform (i.e., retrospective component). Participants could not turn back on the road, so if they had forgotten a PM cue they had to report by “I forgot to stop here.” In the same way if they did not remember the action they had to take once the moment come they had to say for example “I have something to do here/now, but I do not know what.” All participants were encouraged during the experiment to say if they remembered something even at the wrong time. A maximum of 2 points could be awarded for each intention: 1 point if participants stopped the car at the appropriate moment (prospective component score); and 1 point if participants recalled the correct action (retrospective component score). More precisely, concerning EB, for the prospective component, we gave 1 point if the stop was in front of the prospective cue or just after; if participants forgot to stop, we gave 0 point. For the retrospective component, 1 point was given if participants recalled the action (0 if not). If they stopped the car anywhere and correctly recalled the action, they were given 0 for the prospective component and 1 for the retrospective component. Conversely, if they stopped at the right place without recalling the action, 1 point was given for the prospective component only. Concerning TB, for the prospective component, 1 point was given if participants stopped at the right time, 0.5 point if they stopped at ±15 s of the right time. If they stopped the car anytime else, 0 point was given. For the retrospective component, 1 point was given if participants recalled the action (take a mediation).

Each EB condition was scored on a total of 6 points. As the TB score varied according to the time it took participants to complete the task (one intention every 2 min), percentages scores were calculated for this condition. For the sake of comparison, EB scores were also transformed as percentages. The raw scores are displayed in “Supplementary Material”.

### Statistical Analyses

In the first part, in view of our small samples, non-parametric analyzes (Mann–Whitney *U* tests) were conducted for intergroup comparisons. In order to assess the encoding process, we compared cognitively normal older individuals to patients with AD on the number of repetitions needed to learn Link-EB, NoLink-EB and TB intentions. We conducted analyzes for EBPM as well as for TBPM and recall of the prospective and retrospective components of these intentions. Then, we compared recall of the different (1) types of intention (Link-EB vs. NoLink-EB vs. TB), and (2) components (prospective vs. retrospective).

In the second part, we ran intragroup analyses to establish a performance profile for each group. We performed a Wilcoxon signed-rank test to assess pairwise comparisons. First, in the recall of types of intentions and second, in the recall of components.

Then, recall of the prospective and retrospective components was compared in each group for Link-EB and NoLink-EB with Mann–Whitney *U* tests.

Finally, to assess the cognitive substrates of PM decline in AD, we conducted correlations analyses with non-parametric Spearman test between cognition and PM intention types (TB, NoLink-EB, and Link-EB) and components (prospective and retrospective) separately, in the group of AD patients.

## Results

### Intergroup Comparisons of PM Performances

#### Encoding of Link-EB, NoLink-EB and TB Intentions

**FIGURE 2:**
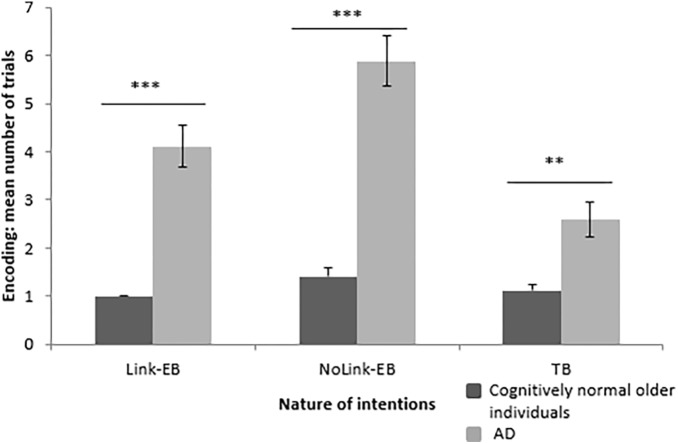
Mean number of trials needed at encoding for each type of intentions (Link-EB vs. NoLink-EB vs. TB) in cognitively normal older adults and patients with AD. EB = event-based, TB = time-based, AD = Alzheimer’s disease. Errors bars represent standard deviation. ^∗∗^ *p* < 0.01. ^∗∗∗^ *p* < 0.001.

An effect of group was found on the number of trials needed to encode each type of intention. Cognitively normal older individuals needed lower number of trials to encode Link-EB intentions (*U* = 15.00, *z* = 4.61, *p* < 0.001, *r* = 0.81), NoLink-EB intentions (*U* = 4.00, *z* = 4.73, *p* < 0.001, *r* = 0.84), and TB intentions (*U* = 52.00, *z* = 3.27, *p* < 0.01, *r* = 0.58) when compared to patients with AD (Figure 2).

#### Recall of Link-EB, NoLink-EB and TB Intentions

**FIGURE 3:**
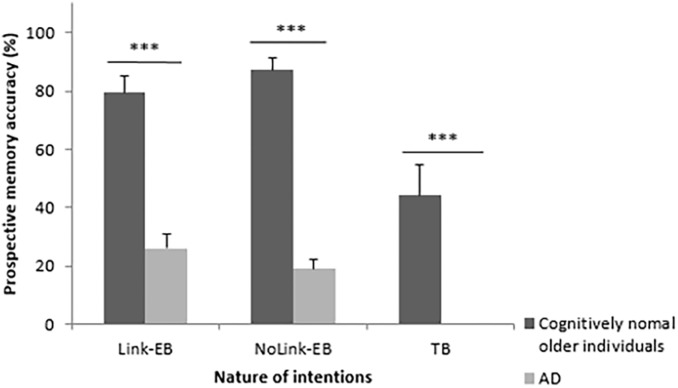
Percentage of correct recall for each type of intentions (Link-EB vs. NoLink-EB vs. TB) in cognitively normal older adults and patients with AD. EB = event-based, TB = time-based, AD = Alzheimer’s disease. Errors bars represent standard deviation. ^∗∗∗^ *p* < 0.001.

An effect of group was found on the recall of each type of intentions. Cognitively normal older individuals recalled more Link-EB intentions (*U* = 15.00, *z* = 4.28, *p* < 0.001, *r* = 0.76), NoLink-EB intentions (*U* = 0.00, *z* = 4.88, *p* < 0.001, *r* = 0.86), and TB intentions (*U* = 59.50, *z* = 3.35, *p* < 0.001, *r* = 0.59) than patients with AD. AD patients had 0% of success for the recall of TB intentions. These results are illustrated in Figure 3.

#### Recall of the Prospective and Retrospective Components

**FIGURE 4:**
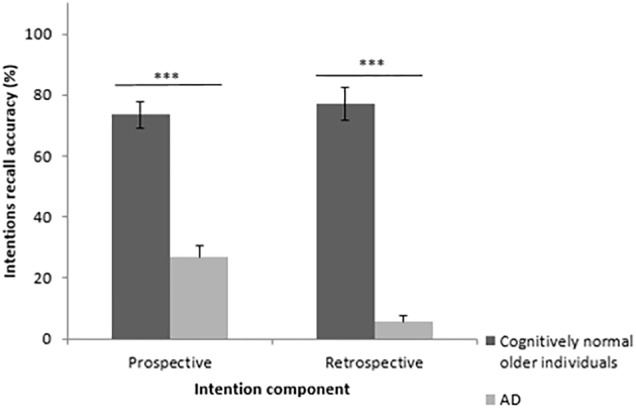
Percentage of correct recall of each component (prospective vs. retrospective) comparing cognitively normal older adults and patients with mild AD. EB = event-based, TB = time-based, AD = Alzheimer’s disease. Errors bars represent standard deviation. ^∗∗∗^ *p* < 0.001.

An effect of group was found on the recall of the prospective and retrospective components. Cognitively normal older individuals recalled more prospective (*U* = 10.00, *z* = 4.45, *p* < 0.001, *r* = 0.79), and retrospective (*U* = 0.50, *z* = 4.89, *p* < 0.001, *r* = 0.86) components than patients with AD. These results are illustrated in Figure 4.

### Intragroup Comparisons of PM Performances

#### Recall of Link-EB, NoLink-EB and TB Intentions in Each Group

Wilcoxon tests showed that cognitively normal older individuals recalled more Link-EB (*z* = 2.20, *p* < 0.05, *r* = 0.42) and NoLink-EB (*z* = 1.73, *p* = 0.01, *r* = 0.60) than TB intentions. There was a trend toward better recall of NoLink-EB intentions than Link-EB intentions (*z* = 1.73, *p* = 0.08, *r* = 0.39) in this group (see Figure 3).

Patients with AD also recalled more Link-EB (*z* = 3.18, *p* < 0.01, *r* = 0.62) and NoLink-EB (*z* = 3.30, *p* < 0.001, *r* = 0.62) than TB intentions. They recalled Link-EB and NoLink-EB intentions at a similar level (*z* = 1.46, *p* = 0.14) (Figure 3).

#### Recall of the Prospective and Retrospective Components in Each Group

Wilcoxon signed-rank test showed that cognitively normal older individuals recalled equally the retrospective than the prospective component of intentions (*z* = 0.35, *p* = 0.73, *r* = 0.07). Conversely, patients with mild AD significantly recalled the prospective component better than the retrospective component (*z* = 3.18, *p* < 0.01, *r* = 0.63) (Figure 4).

### Comparison of the Correct Responses of the Prospective vs. Retrospective Components for Link-EB and NoLink-EB in Each Group

**FIGURE 5:**
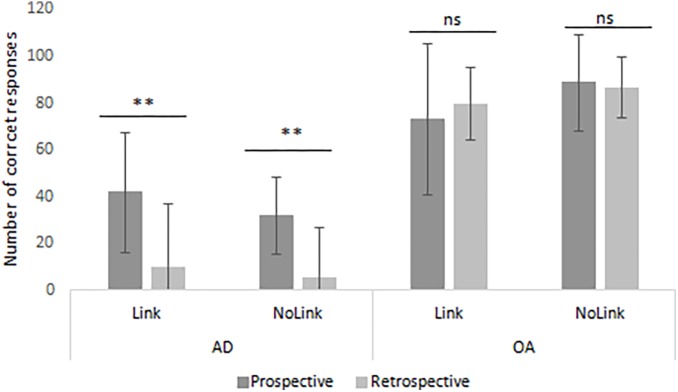
Comparison of the correct responses of the prospective vs. retrospective components of Link-EB and NoLink-EB in each group. EB = event-based, AD = Alzheimer’s disease, OA = older adults. Errors bars represent standard deviation. ^∗^ *p* < 0.05. ^∗∗^ *p* < 0.01. ns = non-significant.

While cognitively normal older individuals recalled both components equally well for Link-EB intentions (z = 1.52, p = 0.13) and NoLink-EB (z = 0.13, p = 0.89), AD patients recalled better the prospective than the retrospective component of Link-EB (z = 2.93, p < 0.01) and NoLink-EB (z = 2.93, p < 0.01) intentions. In each group, there was no difference between the recall of the Link-EB vs. NoLink-EB neither for the prospective component nor for the retrospective component. These results are illustrated in Figure 5.

### Relationships Between PM Intention Types and Cognitive Assessment in AD Patients

**FIGURE 6:**
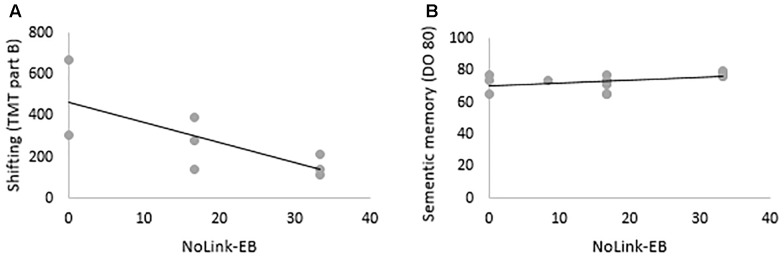
Correlations between **(A)** shifting, **(B)** semantic memory and NoLink-EB intentions in AD patients.

Spearman’s correlations were run between patients’ scores in neuropsychological tests and their performance when recalling Link-EB and NoLink-EB, intentions. TB intentions were excluded from these analyses because there was 0% of success in AD patients. NoLink-EB intentions correlated with shifting tested by TMT part B (*r* = –0.81, *p* < 0.05) and semantic memory tested by DO 80 (*r* = 0.55, *p* < 0.05). No correlation was significant with Link-EB intentions. These results are illustrated in Figure 6.

### Relationship Between Retrospective and Prospective Components and Cognitive Assessment in AD Patients

**FIGURE 7:**
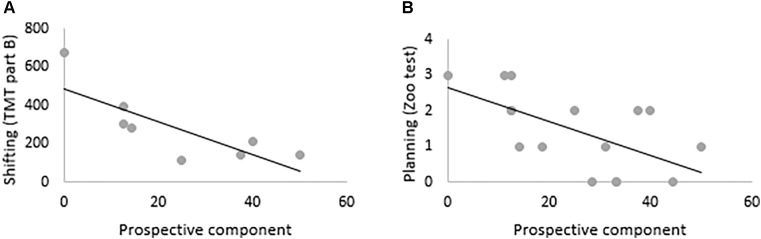
Correlations between **(A)** shifting, **(B)** planning and prospective component in AD patients.

Spearman’s correlations showed that the prospective component correlated with TMT part B (*r* = –0.83, *p* < 0.05) and planning (*r* = –0.63, *p* < 0.01). Regarding the retrospective component, the floor effect observed in the AD group prevented the realization of correlations. Results of correlations for the prospective component are displayed in Figure 7.

## Discussion

We conducted the present study to investigate the effects of mild AD on PM. Participants were asked to recall seven intentions in a virtual environment, allowing them to benefit from a more ecological assessment while keeping a tight experimental control on measurements. Our findings reinforce those of previous studies and bring new insights into the nature of PM impairment in mild AD. First, PM impairment was expressed regardless of the type of intentions (i.e., Link-EB, NoLink-EB or TB), with the strongest difficulties for TB intentions. Furthermore, while previous findings suggested that patients with mild AD exhibit comparable impairments in the recall of both components of intentions (Jones et al., 2006), our results indicate that the recall of the retrospective component is more challenging than that of the prospective component in early AD.

### Prospective Memory in Patients With Mild AD

#### Impaired Recall of EB and TB Intentions

As we expected, patients with AD had difficulties right from the encoding phase and especially for NoLink-EB intentions. This result was not surprising because the episodic memory encoding is particularly impaired at the beginning of AD and the encoding in PM uses relatively similar or even more complex processes. Moreover, they were impaired in the recall of Link-EB, NoLink-EB and TB intentions. Whereas they were able to recall a few Link-EB and NoLink-EB intentions, none of them recalled any TB intention. This finding is in line with previous studies showing that the recall of TB intentions is the most challenging for patients with mild AD (Dermody et al., 2016). In the present study, in order to recall TB intentions at the right moment, individuals had to self-initiate clock checking with the clock not being embedded in the virtual environment. As a consequence, if participants wanted to check the clock while monitoring the virtual environment to detect prospective cues, they regularly had to shift the focus of their attention from the virtual environment to the clock, and vice versa. During the experiment, we observed that patients with AD never engaged in strategic clock-checking, which can suggest that they were not able to focus on more than one task at a time (Maylor et al., 2002). In our study, AD patients seemed to have forgotten the TB intention. Interestingly, when they were asked to report the strategies they had used during the task at the end of the experiment, cognitively normal older individuals reported that they had focused primarily on TB intentions, because they had anticipated difficulty in recalling time-related intentions. AD patients did not report the use of such a strategy. In summary, AD patients seem to present both encoding and retrieval impairment in PM.

#### Impairment Regardless of the Strength of the Link Between the Prospective Cue and the Retrospective Component

We expected cognitively normal older individuals to perform better when there was a strong link between the prospective cue and the retrospective component. Surprisingly, they tended to recall more NoLink-EB than Link-EB intentions. However, they initially repeated more NoLink-EB intentions than Link-EB to encode the action, which reflects their awareness of the difficulty of the task. They also reported in debriefing questionnaires (data not shown) that they had been helped by the incongruence of the NoLink-EB intentions to increase their memorization Thus, those strategies explained these unexpected results. By contrast, AD patients were equally impaired when recalling Link-EB and NoLink-EB intentions, in line with previous studies (Maylor et al., 2002; Dermody et al., 2016).

Impaired recall of NoLink-EB intentions was expected because remembering these intentions requires the involvement of executive/controlled processes in addition to retrospective memory (Guynn et al., 1998; Marsh et al., 2003; McDaniel et al., 2004). Interestingly, they did not recall Link-EB intentions more efficiently which suggests that they did not benefit from strong associations. This finding corroborates those of Maylor et al. (2002), which showed that the deleterious effect of AD on the recall of intentions is not reduced when the prospective cue and retrospective component are strongly associated, that is, when relying on automatic processes should be sufficient to retrieve intentions. From a process-oriented standpoint, this finding suggests an inability either to detect a prospective cue or to spontaneously associate a prospective cue with an intention. Since the prospective cues were arranged so that they could be detected extremely easily in the virtual city, it is more likely that they did not elicit spontaneous retrieval owing to impairment of these automatic processes. This finding is consistent with the AD-related impairment of medial temporal structures that are known to support these mnemonic automatic processes (La Joie et al., 2014).

To our knowledge, research works studying PM in AD did not assess the relationship between PM decline and other cognitive functions. In our study, correlation analysis showed that NoLink-EB decline was linked to difficulties in semantic memory and shifting. These results are consistent with previous studies, in cognitively normal older individuals, showing that memory decline has deleterious effect on EBPM (Gonneaud et al., 2011), notably in the NoLink condition (Lecouvey et al., 2017). The role of semantic memory in PM retrieval has not really been described in previous studies but we suggest that it is involved in the creation of links between the prospective cue and the action to perform when these two elements are weakly associated. This link is determinant in order to memorize the intention and recognize the prospective cue in the right conditions. Regarding shifting, in the context of our task, it may allow to move attention from driving to detecting the cue to retrieve the intention at the right moment.

#### Impaired Recall of Both Components, Especially the Retrospective One

Another main finding of this study is that, besides the fact that the recall of both the prospective and retrospective components was found impaired in patients, they encountered greater difficulty recalling the retrospective component than the prospective one. This finding is not in line with two previous studies showing fairly similar impairment of the recall of both components (Jones et al., 2006; Costa et al., 2015) but is not counterintuitive as the recall of retrospective component is thought to rely primarily on retrospective episodic memory (Hainselin et al., 2011), which is dramatically impaired in AD. Interestingly, when they correctly recalled the prospective component of EB intentions, and gave an answer for the retrospective component, patients with AD tended to provide a prototypical but incorrect action (e.g., “at the newsstand, I buy a newspaper” instead of “I buy a TV program”).

Even less prominent, patients with AD demonstrated an impairment in the recall of the prospective component, which is usually described as being primarily supported by executive functions (Martin et al., 2003) known to be also affected in the earliest stages of AD (Fouquet et al., 2009). The recall of the prospective component was correlated with executive performance, more precisely with shifting and planning functions that are essential to complete a PM task (Schnitzspahn et al., 2013), that is in our study, to stop the car at the right moment.

### Virtual Reality in Patients With AD

This study demonstrates the relevance of VR-based tasks for assessing PM in patients with AD. Our decision to use such an immersive virtual environment was based on the knowledge that it represents a good compromise between naturalistic and laboratory tasks, allowing complex naturalistic situations to be reproduced while maintaining a high level of experimental control (Knight and Titov, 2009). In our study, VR allowed a more accurate and realistic assessment of PM. Thus, VR not only allows the assessment of cognitive impairments, but also provides insights into their functional repercussions in real life (Déjos et al., 2012). Nevertheless, although the present study shows that VR is a relevant way of assessing cognitively impaired individuals, the use of this technique can lead to difficulties in fragile patients if special precautions are not taken. Debriefing data (data not shown) suggested, for example, that patients had greater difficulty adapting to the equipment during the familiarization phase, which may have led to increased anxiety, thus interfering with the recall of intentions. In order to minimize this effect, participants solely used pedals to navigate through the virtual city. Moreover, the length of the familiarization phase was adjusted so that participants only discovered the virtual city once they felt entirely comfortable with the equipment. This phase was significantly longer for patients (data not shown), probably due to their cognitive impairment plus the fact that some of them had given up driving for many years in real life. Various cognitive disorders affecting procedural learning and causing disorientation may also have slowed down patients. Despite all these provisos, the VR assessment remained possible, and all the participants described the VR immersion as a pleasant experience. They completed the task in conditions that allowed us to collect reliable data, which is a promising sign for forthcoming experiments.

### Limitations and Future Directions

Some limitations are present in this study. First, although we obtained large size effects suggesting that results are generalizable to the general population, future studies should be realized with a higher number of participants.

Regarding the PM task, the low number of PM intentions per conditions constitutes a limit. PM performance was assessed with only seven intentions to recall. However, it is important to note that this number of intentions was already challenging for mild AD participants. Increasing the number of intentions should thus be an issue, but focusing on some specific processes in future studies might help reduce power issues (e.g., assessing only EB-link intentions). Another problem in this study was the 0% of success in AD patients for TB intention that could be due to difficulties in time monitoring strategies or even to a complete forgetfulness of this intention. Future study will have to address this question, designing experiments focusing more specifically on TB intentions.

Finally, the protocol did not include control posttests to assess the maintaining of intentions after the task in participants and did not include any questionnaire about immersive experience. As a result we cannot further assess the potential association between immersion and performance.

## Conclusion

This study is the first to have explored the effects of mild AD on PM using a VR task. Findings confirm impaired recall of both prospective and retrospective components of EB and TB intentions in mild AD who were more efficient when recalling the prospective vs. retrospective component of intentions. This last result suggests that early episodic memory impairment has a greater impact on the recall of retrospective component. Our findings encourage the development of new tools to identify impairments in patients with mild AD and to investigate their residual functional abilities. The ecological validity of the study still needs to be improved with longer and more realistic tasks. These tools would also be useful for providing individualized therapeutic interventions to help maintain patients’ wellbeing and safety in everyday life. Our findings suggest the need to provide support mainly for the recall of the retrospective component (e.g., use of lists) but also for the prospective one (e.g., schedule reminders).

## Author Contributions

GL was involved in this study from design to drafting. GL and AM conducted all the statistical analyses and played a central role in interpreting the results and writing the article. AP, LFDS and VdLS participated in the acquisition of clinical and cognitive data, and especially in the careful screening of our cohort. EO contributed to the elaboration of the task in VR. JG, FE, and PP provided their critical revision of the manuscript. BD supervised and coordinated the team work from start to finish.

## Conflict of Interest Statement

The authors declare that the research was conducted in the absence of any commercial or financial relationships that could be construed as a potential conflict of interest.
