# Supplementary figures and images for: An Impairment of Prospective Memory in Mild Alzheimer’s Disease: A Ride in a Virtual Town
Source: Front Psychol. 2019 Feb 12;10:241. doi: 10.3389/fpsyg.2019.00241 (PMC6379453; doi:10.3389/fpsyg.2019.00241)

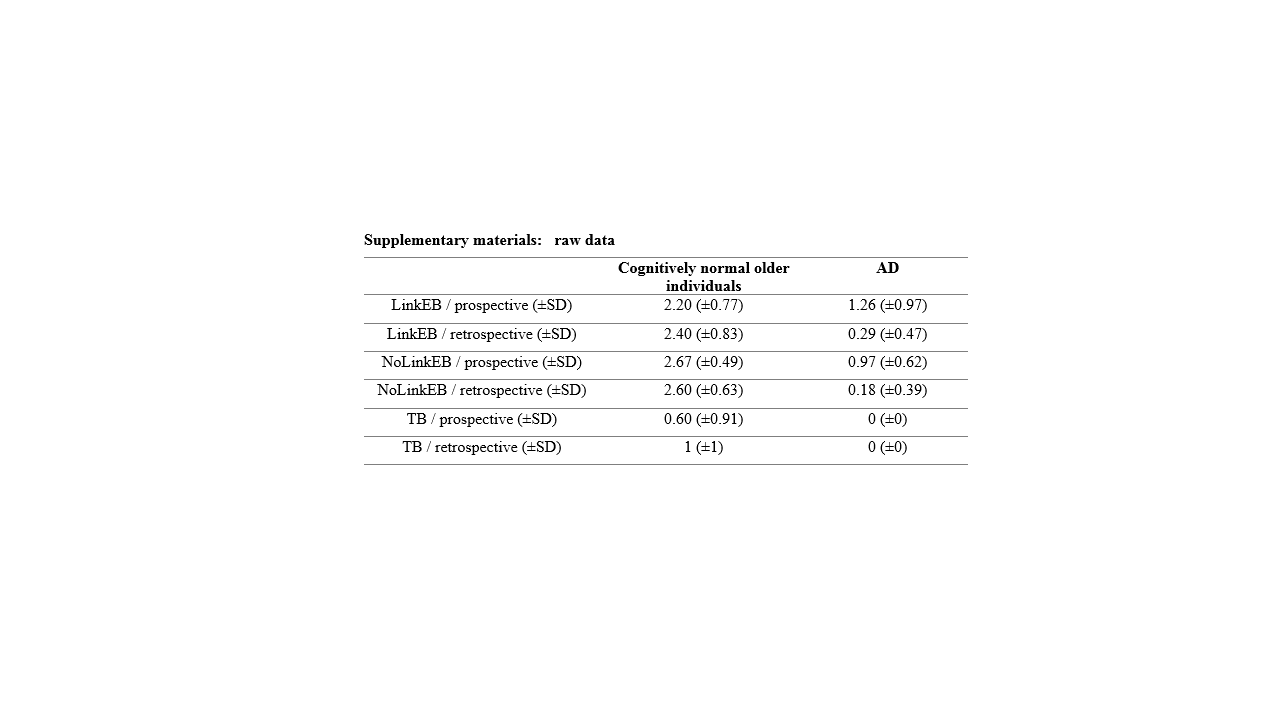

Supplement: Supplementary file 1 [file Image_1.TIF]
